# Uncovering cargo clients and accessory factors of AP-1 and AP-4 through vesicle proteomics

**DOI:** 10.1073/pnas.2508961122

**Published:** 2025-10-01

**Authors:** Ziqing Peng, Jingran Fan, Yang Liu, Qinyu Jia, Junkun An, Jianying Wang, Yan Huang, Zhong-Ping Yao, Yusong Guo

**Affiliations:** ^a^Division of Life Science and State Key Laboratory of Molecular Neuroscience, The Hong Kong University of Science and Technology, Hong Kong, Hong Kong; ^b^State Key Laboratory of Chemical Biology and Drug Discovery, Research Institute for Future Food, Research Centre for Chinese Medicine Innovation, and Department of Applied Biology and Chemical Technology, The Hong Kong Polytechnic University, Hung Hom, Kowloon, Hong Kong, Special Administrative Region, China; ^c^State Key Laboratory of Chinese Medicine and Molecular Pharmacology (Incubation), and Shenzhen Key Laboratory of Food Biological Safety Control, Hong Kong Polytechnic University Shenzhen Research Institute, Shenzhen 518057, China; ^d^Thrust of Bioscience and Biomedical Engineering, Hong Kong University of Science and Technology, Guangzhou 511453, China

**Keywords:** cargo sorting, vesicular trafficking, adaptor protein complexes, *trans*-Golgi network, the secretory pathway

## Abstract

The *trans*-Golgi network (TGN) is a crucial sorting station in the secretory pathway, where adaptor protein (AP) complexes ensure selective cargo packaging into transport vesicles. However, the complete repertoire of cargoes and regulators associated with individual AP complexes remains poorly defined. Intriguingly, AP-4-mediated TGN export operates independently of clathrin, suggesting the involvement of uncharacterized accessory factors in vesicle biogenesis. To address these gaps, we developed an in vitro vesicle formation assay using wild-type HeLa cells or cells deficient in AP1γ1 or AP4ε, reconstituting their roles in packaging their known clients, Vangl2 and ATG9A, respectively. Coupling this assay with label-free quantitative mass spectrometry, we mapped distinct cargo profiles for AP-1 (which buds from the TGN and ARF1-positive endosomes) and AP-4, identifying the 45 kDa calcium-binding protein (CAB45) as an AP-1-dependent cargo and the Type-1 angiotensin II receptor-associated protein (ATRAP) as an AP-4-dependent cargo. Additionally, we uncovered PRRC1 and WDR44 as cytosolic regulators essential for AP-4-mediated TGN export. Our study advances the mechanistic understanding of AP-1 and AP-4 in secretory trafficking and provides a robust strategy to systematically identify cargo clients and accessory factors for specific adaptor complexes.

## Significance

Adaptor protein complexes AP-1 and AP-4 mediate protein sorting at the *trans*-Golgi network (TGN), yet their cargo clients and regulators remain incompletely defined. Here, using a large-scale vesicle formation assay coupled to label-free quantitative mass spectrometry, we delineate distinct cargo repertoires for the two adaptors, identifying CAB45 as an AP-1–dependent cargo and ATRAP as an AP-4–dependent cargo. We also uncover PRRC1 and WDR44 as cytosolic regulators of AP-4–mediated TGN export. Together, these results expand our understanding of adaptor function and specificity, offer insights into mechanisms of cargo sorting and vesicular trafficking, and provide a robust framework for systematically discovering additional cargo clients and regulatory factors within the secretory pathway.

The *trans*-Golgi network (TGN) serves as a critical transport hub in the secretory pathway, ensuring the accurate delivery of cargo proteins to their respective destinations. To maintain the fidelity of protein transport, the TGN employs sophisticated protein sorting machineries that package cargo into specific transport vesicles for delivery to downstream compartments. Defects in TGN sorting disrupt protein targeting, leading to a range of physiological impairments, including loss of cell polarity, compromised immunity, and dysregulated secretion (1).

Among the key players in protein sorting are the adaptor protein complexes (APs), which mediate cargo selection and vesicle formation in both exocytic and endocytic pathways (1). In higher eukaryotes, five AP complexes have been identified, each composed of two large subunits, a medium subunit, and a small subunit. Notably, AP-1, which functions at the TGN and ARF1-positive endosomes, and AP-4 are particularly important for cargo export from the TGN. Mutations in subunits of these complexes are linked to severe human diseases. For instance, mutations in the AP1S1 gene, encoding the σ1 subunit of AP-1, are associated with MEDNIK syndrome (2), while mutations in AP1S2 cause X-linked mental retardation (3, 4). Similarly, mutations in any subunit of AP-4 result in “AP-4 deficiency syndrome,” a complex form of hereditary spastic paraplegia (HSP) (5). Additionally, mutations in the ε subunit of AP-4 are linked to familial persistent stuttering 1 (STUT1).

Understanding the cargo clients of AP-1 and AP-4 is essential for elucidating their functional roles in normal physiology and disease. To date, AP-1 has been shown to mediate the TGN export of diverse cargoes, including the planar cell polarity protein Vangl2, which is essential for establishing cell polarity (6), and potassium channel Kir2.1 (7). AP-1 also plays a key role in delivering basolateral cargo proteins to maintain apical-basolateral polarity (8, 9) and sorting of dendritic proteins at the TGN in neuronal cells (10). On the other hand, AP-4 regulates the export of autophagy-related protein 9A (ATG9A) from the TGN, facilitating autophagosome formation (11), and mediates the transport of Apolipoprotein E receptor 2 (ApoER2), which is crucial for central nervous system development and function (12). AP-4 is also shown to mediate TGN export of Amyloid Precursor Protein, thereby reducing γ-secretase-mediated cleavage to amyloid-β peptide (13). Despite these advances, the full repertoire of cargoes and accessory factors involved in AP-1- and AP-4-mediated trafficking remains poorly understood. Notably, AP-4-mediated TGN export appears to operate independently of clathrin, suggesting the involvement of unidentified accessory factors in vesicle formation.

To comprehensively understand the functional roles of adaptor proteins, robust approaches are needed to systematically identify their cargo clients and the cytosolic factors essential for adaptor protein complex-mediated TGN export. Previous studies have employed various strategies to address this challenge. For example, the PAIRS (Pairing Analysis of Cargo Receptors) imaging approach identified cargo proteins dependent on specific receptors for ER export in yeast, though it was limited to analyzing around 150 fluorescently tagged cargo molecules (14). Another method, dynamic organellar mapping combined with proteomics, revealed AP-4 cargo clients such as ATG9A, SERINC1, SERINC3, and DAGLB (15, 16). However, this approach assessed protein abundances within organelles rather than directly on vesicles.

To overcome these limitations, we recently developed a large-scale vesicle formation assay coupled with label-free quantitative mass spectrometry (17), allowing direct analysis of vesicle-associated proteins. In this study, we used cytosol from AP1γ1 or AP4ε knockout (KO) cells to perform the vesicle formation assay, isolated vesicle fractions, and analyzed their contents via quantitative mass spectrometry. This strategy enabled us to identify AP-1- and AP-4-dependent cargo proteins for TGN export, as well as key cytosolic factors required for AP-4-mediated trafficking. Our findings provide insights into the roles of AP-1 and AP-4 and uncover cytosolic components critical for AP-4-dependent TGN export.

## Results

### An In Vitro Vesicle Formation Assay Combined With Proteomic Analysis to Identify the Cargo Clients of AP-1.

**Fig. 1.**
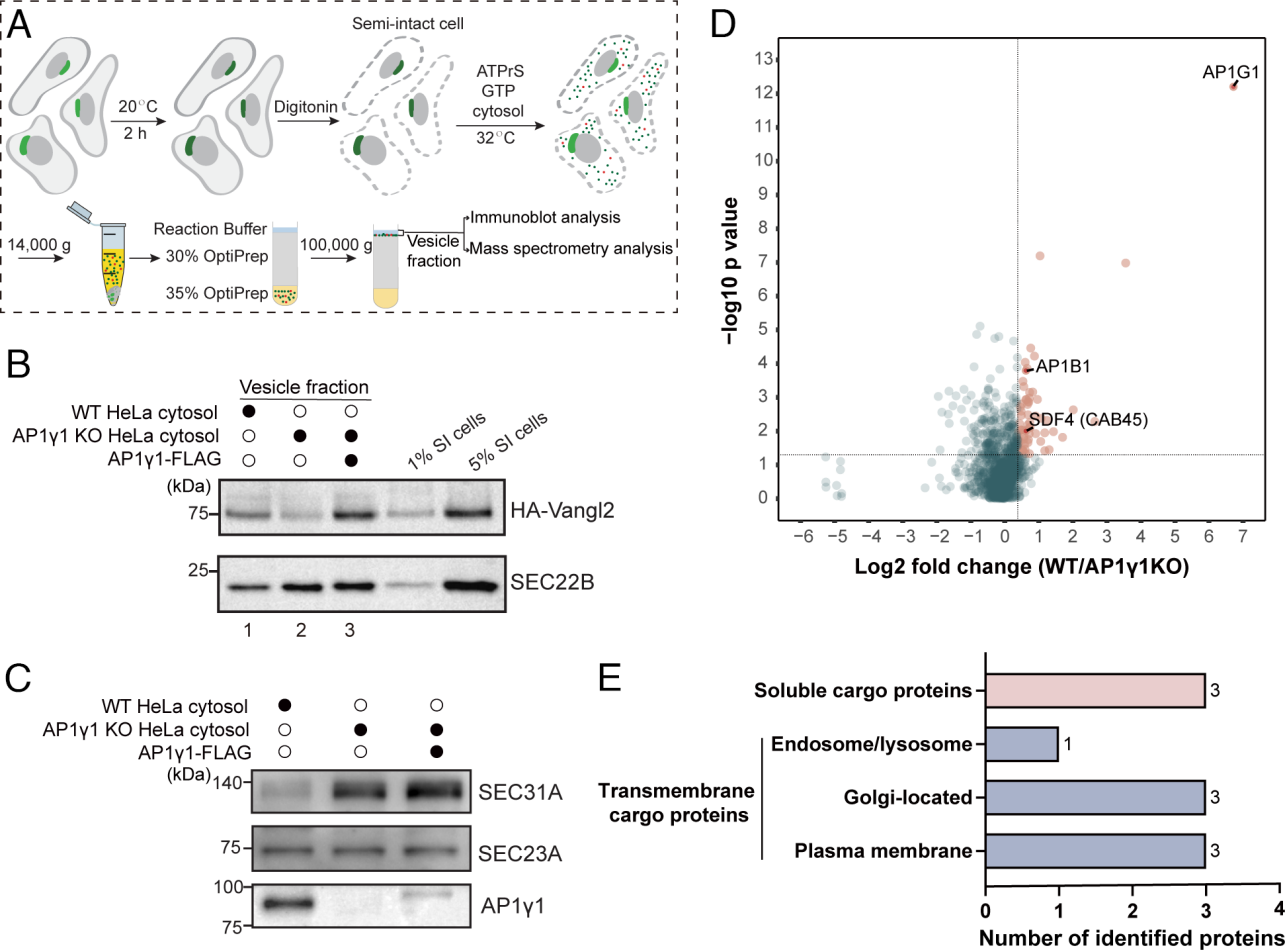
Vesicle proteomics to identify AP-1 cargo clients. (*A*) Schematic of the in vitro vesicle formation assay. (*B*) Reconstitution of the packaging of HA-Vangl2 into transport vesicles. Assays were performed using cytosol from WT HeLa cells, AP1γ1 KO HeLa cells, or AP1γ1 KO HeLa cells expressing AP1γ1-FLAG. (*C*) The abundances of AP1γ1, COPII components SEC31A and SEC23A in indicated HeLa cytosol were analyzed by immunoblot. (*D*) Proteomic analysis of vesicle fractions. Donor membranes from AP1γ1 KO HeLa cells were incubated with cytosol from WT or AP1γ1 KO cells. Protein abundance (log2 fold change, WT vs. KO) is plotted against significance (−log10 *P* value). (*E*) Distribution of identified cargo proteins by subcellular localization predicted by UniProt. SI cells, semi-intact cells; ATP regeneration system (ATPrS).

To uncover cargo proteins that rely on AP-1 or AP-4 for packaging into transport vesicles, we utilized a well-established in vitro vesicle formation assay (17), as shown in Fig. 1*A*. Briefly, cells were incubated at 20 °C to accumulate newly synthesized cargo proteins at the TGN. After permeabilization with digitonin and subsequent washing to remove cytosolic proteins, the semi-intact cells (SI cells) were incubated at 32 °C with cytosol, GTP, and an ATP regeneration system (ATPrS). Following incubation, newly formed vesicles were separated from the donor membranes by centrifugation. The resulting supernatant, containing the vesicles, was adjusted to 35% Opti-Prep, overlaid with 30% Opti-Prep and reaction buffer, and subjected to centrifugation to separate the vesicles from unassociated cytosolic proteins.

As an initial step, we utilized this assay to reconstitute the packaging of Vangl2, a known AP-1 cargo client, into transport vesicles. Vangl2 is critical for regulating planar cell polarity, and its export from the TGN depends on a tyrosine sorting motif that is recognized by AP-1 (6). We found that cytosol prepared from wild-type (WT) HeLa cells, but not from AP1γ1 KO HeLa cells, facilitated the packaging of HA-Vangl2 into transport vesicles (Fig. 1*B*, compare lanes 1 and 2). Furthermore, the reduced budding efficiency observed with AP1γ1 KO cytosol was rescued by using cytosol derived from AP1γ1 KO HeLa cells overexpressing AP1γ1-FLAG (Fig. 1*B*, compare lanes 2 and 3). In contrast, SEC22B, a standard cargo protein associated with COPII vesicles, exhibited higher budding efficiency when using AP1γ1 KO HeLa cytosol compared to WT HeLa cytosol, regardless of the expression of exogenous AP1γ1-FLAG (Fig. 1*B*). We found that the abundances of the inner COPII coat subunit, SEC23A, remained comparable between the cytosol prepared from WT and AP1γ1 KO HeLa cells, while the abundance of the outer COPII coat subunit, SEC31A, was markedly elevated in the KO cytosol (Fig. 1*C*). Notably, reintroducing AP1γ1-FLAG did not restore SEC31A to WT levels (Fig. 1*C*). This observation is consistent with the increased abundance of SEC22B in vesicle fractions when using AP1γ1 KO HeLa cytosol, even in the presence of exogenous AP1γ1-FLAG (Fig. 1*B*).

The successful reconstitution of Vangl2 packaging validates the functionality of our vesicle formation assay and implicates its potential for identifying AP-1-dependent cargo proteins. Building on this, we performed a large-scale vesicle formation assay combined with label-free quantitative mass spectrometry to systematically identify AP-1 cargo clients. Using semi-intact cells derived from AP1γ1 KO HeLa cells and cytosol extracted from either WT or AP1γ1 KO HeLa cells, we identified 1,205 proteins in the vesicle fraction (Dataset S1, Sheet 1). For each protein, we calculated the fold change of the abundance in the WT group compared to the AP1γ1 KO group. This analysis revealed 66 proteins with a fold change greater than 1.3 and a p-value less than 0.05 (Fig. 1*D*, highlighted in red dots; Dataset S1, Sheet 2). Importantly, two subunits of the AP-1 complex were among the identified proteins, validating the reliability of our approach.

Of the 66 proteins identified, 6 proteins were annotated as transmembrane cargo proteins predicted by UniProt, and 3 were soluble secretory cargo proteins (Fig. 1*E* and Dataset S1, Sheet 3). Among the predicted transmembrane proteins, 3 were predicted to show Golgi localizations, 3 were predicted to show plasma membrane localizations, and 1 was predicted to show endosomal and lysosomal localizations (Fig. 1*E* and Dataset S1, Sheet 3).

### CAB45 Is a Cargo Client of AP-1.

**Fig. 2.**
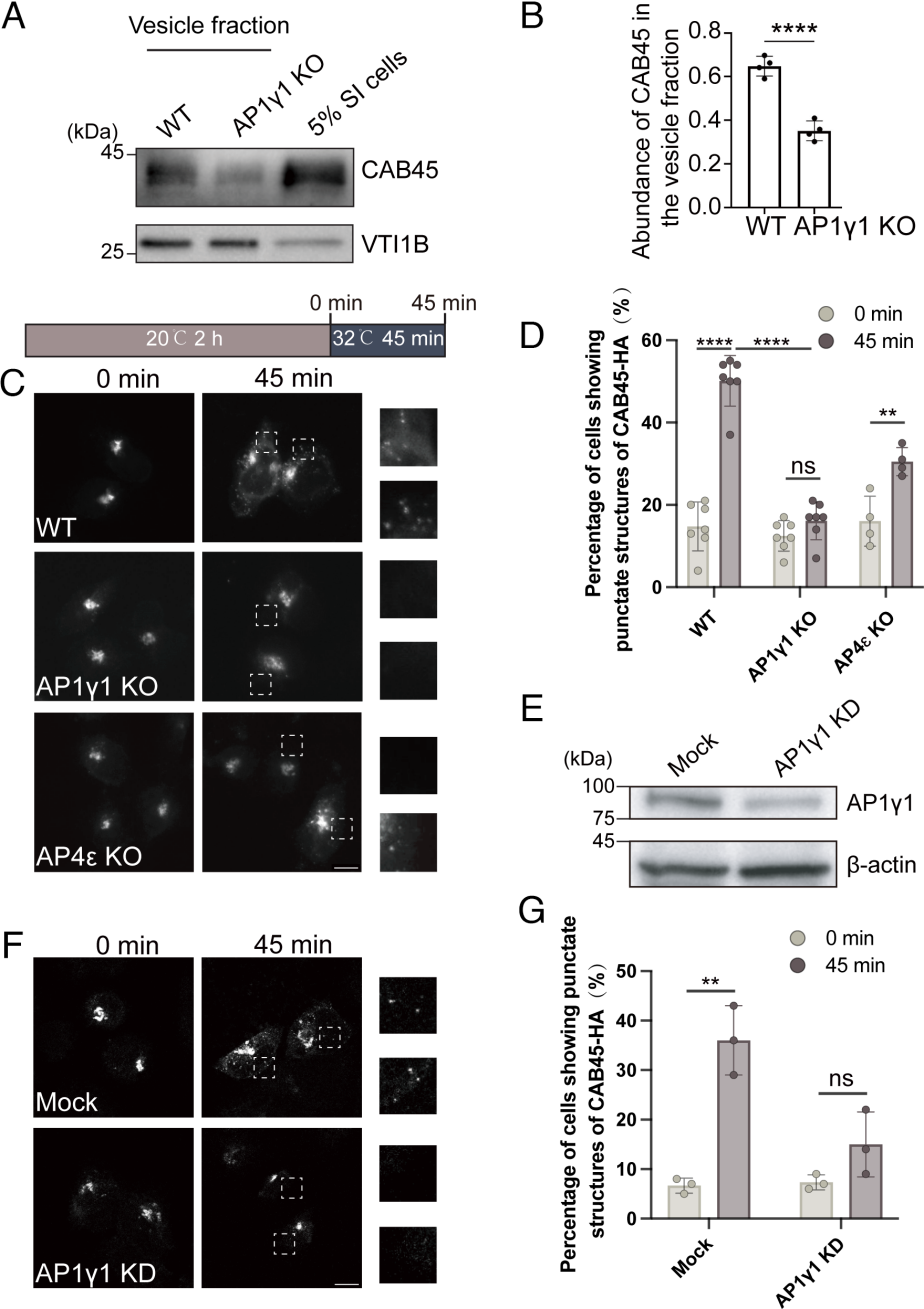
AP-1 mediates the packaging of CAB45 into TGN-derived vesicles. (*A* and *B*) The vesicle formation assay was performed using cytosol from WT HeLa cells or AP1γ1 KO HeLa cells. The vesicle fraction was analyzed by immunoblot (*A*). The abundances of CAB45 in vesicles normalized to VTI1B were quantified (*B*, n = 4, mean ± SD). For each replicated experiment, the sum of the normalized abundance was normalized to 1. (*C*–*G*) WT, AP1γ1 KO, or AP4ε KO HeLa cells were transfected with plasmids encoding CAB45-HA (*C*). HeLa cells were transfected with control siRNA (Mock) or AP1γ1 siRNA (AP1γ1 KD), followed by CAB45-HA expression (*F*). After cycloheximide treatment (3 h) and 20 °C block (2 h), cells were shifted to 32 °C for 0 or 45 min, and the localizations of the indicated proteins were analyzed (*C* and *F*). (Scale bar, 10 μm.) The abundances of AP1γ1 and β actin in cell lysates were analyzed by immunoblot (*E*). Percentages of cells showing punctate structures of CAB45-HA in each experimental group were quantified (*D* and *G*, n ≥ 3, mean ± SD). Each dot represents one independent experiment, with 47 to 166 cells quantified per group in each replicate. Statistical significance was determined using an unpaired *t* test; ****P < 0.0001; **P < 0.01; ns, no significant. SI cells, semi-intact cells.

We then focused our analyses on one of the identified cargo clients located at the TGN lumen, CAB45 (Fig. 1*D*), and investigated whether AP-1 mediates its export from the TGN. Consistent with our proteomic data, immunoblot analysis of the vesicle fraction revealed that the packaging of CAB45 into vesicles was significantly reduced when using cytosol from AP1γ1 KO cells compared to cytosol from WT cells (Fig. 2 *A* and *B*). In contrast, the packaging of VTI1B remained unaffected by AP1γ1 depletion (Fig. 2*A*).

To further confirm that TGN export of CAB45 is mediated by AP-1, we employed a temperature shift assay. In this experiment, cells expressing CAB45-HA were first incubated at 20 °C for 2 h to block cargo protein export from the TGN, followed by a shift to 32 °C to release the cargo. After incubation at 20 °C, CAB45-HA primarily accumulated at the TGN in WT, AP1γ1 KO, and AP4ε KO HeLa cells, with fewer than 20% of cells displaying punctate structures (Fig. 2 *C* and *D*). Following the temperature shift to 32 °C for 45 min, CAB45-HA was efficiently released from the Golgi in WT and AP4ε KO HeLa cells. However, in AP1γ1 KO HeLa cells, CAB45-HA remained predominantly accumulated at the Golgi in most cells (Fig. 2*C*). The percentage of cells with punctate CAB45 localization after temperature shift was significantly lower in AP1γ1 KO cells than in WT cells (Fig. 2*D*), indicating a defect in CAB45 export from the TGN in the absence of AP1γ1. Similarly, WT HeLa cells transfected with siRNA targeting AP1γ1 also exhibited defects in CAB45-HA export from the TGN (Fig. 2 *E*–*G*). These findings demonstrate that AP-1 plays a critical role in regulating the TGN export of CAB45, establishing it as an AP-1-dependent cargo protein.

After the temperature shift, a significant amount of CAB45 remains localized in the Golgi region of WT cells (Fig. 2 *C* and *F*). This finding is consistent with a previous study (18), which reported that only 17% of CAB45 is released into the medium after a 2-h incubation at 37 °C, following a 2-h incubation at 20 °C. One possible explanation is that, unlike canonical secretory cargoes, CAB45 maintains a steady-state localization within the Golgi despite being capable of secretion. As a result, its export efficiency may inherently differ from that of typical soluble secretory proteins.

### An In Vitro Vesicle Formation Assay Combined With Proteomic Analysis to Identify the Cargo Clients of AP-4.

**Fig. 3.**
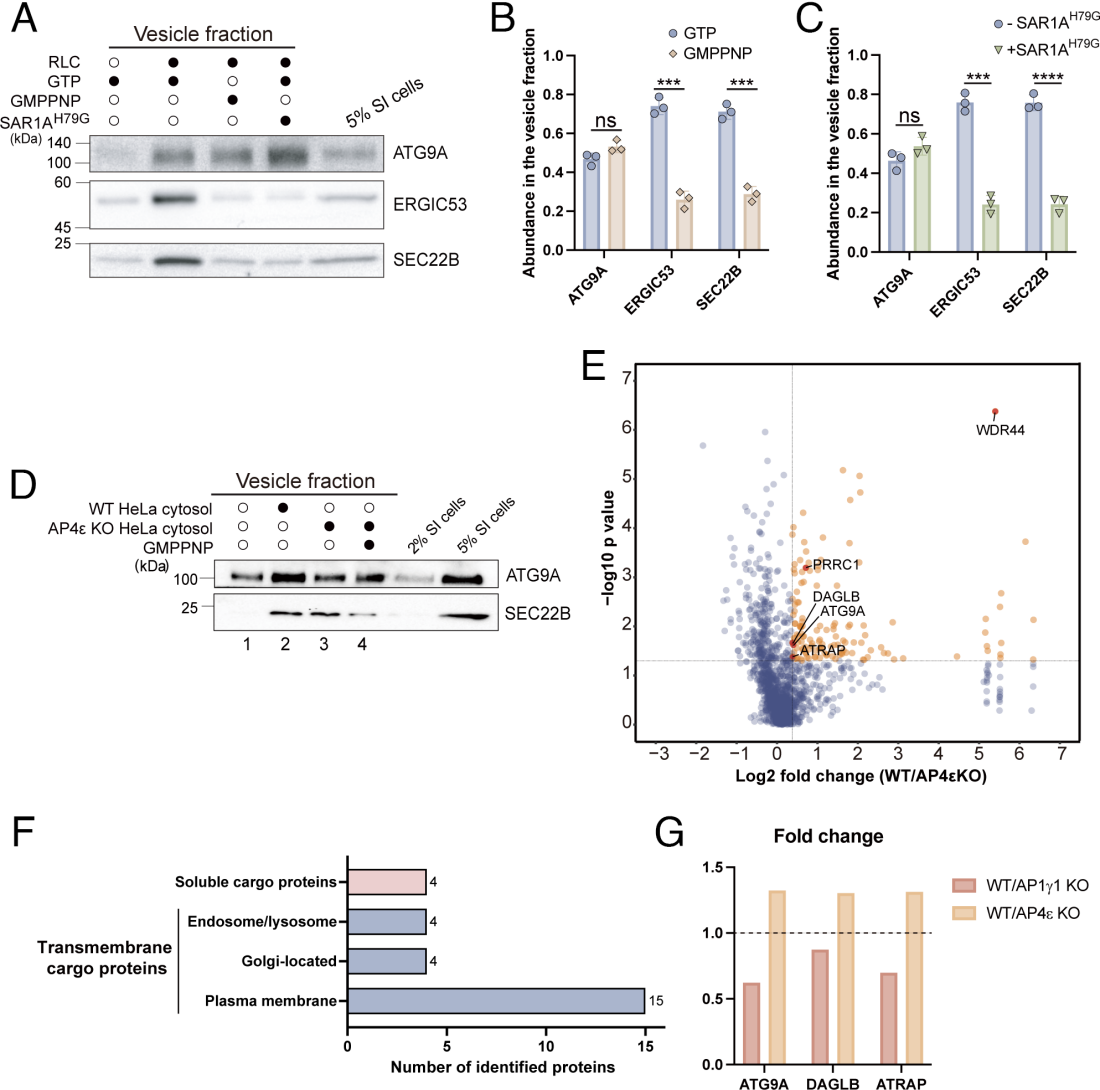
Vesicle proteomics to identify AP-4 cargo clients. (*A*–*C*) The vesicle formation assay was performed using the indicated reagents. The vesicle fractions were then analyzed by immunoblot (*A*) and the abundance of indicated proteins were quantified (*B* and *C*, n = 3, mean ± SD). For quantification analysis, the sum of the abundances of the indicated proteins in the GTP and GMPPNP groups (*B*) or in the plus SAR1A^H79G^ and minus SAR1A^H79G^ (*C*) was normalized to 1. (*D*) The vesicle formation assay was performed, and the vesicle fractions were analyzed by immunoblot. (*E*) The vesicle formation assay was performed using donor membranes from AP4ε KO HeLa cells and cytosol from WT or AP4ε KO cells. The proteins in the vesicle fractions were analyzed by label-free quantitative mass spectrometry, and the protein abundance (log2 fold change, WT vs. AP4ε KO) was plotted against significance (−log10 *P* value). (*F*) Distribution of identified cargo proteins by subcellular localization predicted by UniProt. (*G*) Fold change (WT/AP1γ1 KO vs. WT/AP4ε KO) of the abundances of the indicated proteins. RLC, rat liver cytosol; SI cells, semi-intact cells. Statistical significance was determined using an unpaired t test; ****P < 0.0001; ***P < 0.001; ns, no significant.

The above analyses demonstrate that the vesicle formation assay, combined with label-free quantitative mass spectrometry, is a robust and effective method for identifying AP-1 cargo proteins. Building on this approach, we next sought to identify cargo proteins that rely on another TGN-localized adaptor, AP-4, for enrichment into TGN-derived vesicles. ATG9A, a known cargo of AP-4, has been reported to interact directly with AP-4 via a tyrosine motif in its cytosolic tail (11). To test whether the vesicle formation assay can be used for identifying AP-4 cargo, we employed this assay to reconstitute the packaging of ATG9A into transport vesicles (Fig. 3*A*). The standard COPII cargo proteins ERGIC53 and SEC22B were used as controls. The packaging of ATG9A, ERGIC53, and SEC22B into vesicles was shown to be cytosol-dependent. Consistent with previous reports, the inclusion of a nonhydrolyzable form of GTP (GMPPNP) or a GTPase-defective mutant of SAR1A (SAR1A^H79G^) inhibited the packaging of ERGIC53 and SEC22B into vesicles (Fig. 3 *A*–*C*). In contrast, the packaging of ATG9A into transport vesicles was not significantly affected by GMPPNP or SAR1A^H79G^ (Fig. 3 *A*–*C*). These results suggest that the release of ATG9A into transport vesicles is independent of GTP hydrolysis, distinguishing its packaging mechanism from that of normal COPII cargo proteins.

We conducted a TGN vesicle formation assay using semi-intact cells derived from AP4ε KO HeLa, along with cytosol from either WT or AP4ε KO HeLa cells (Fig. 3*D*). The cytosol from WT HeLa cells, but not from AP4ε KO HeLa, enhanced the packaging of ATG9A into transport vesicles (Fig. 3*D*, compare lanes 1 to 3). This finding confirms that the vesicle formation assay effectively recapitulates the involvement of AP-4 in packaging of ATG9A into transport vesicles. To identify AP-4 cargo clients, we performed the vesicle formation assay paired with label-free quantitative mass spectrometry analysis, using semi-intact cells from AP4ε KO HeLa and cytosol extracted from WT or AP4ε KO HeLa (Fig. 3*E*). Our analysis in the vesicle fraction identified a total of 1,451 proteins (Dataset S2, Sheet 1), among which 139 demonstrated a significant change with a fold increase over 1.3 and a p-value less than 0.05 (Fig. 3*E*, highlighted in orange dots, Dataset S2, Sheet 2). Notably, this group includes two known AP-4 cargos, ATG9A and DAGLB (11, 16), confirming the efficacy of our approach in uncovering AP-4 cargo clients. Of these 139 proteins, 18 were predicted to be transmembrane proteins, and 4 were soluble secretory cargo proteins (Fig. 3*F* and Dataset S2, Sheet 3). Among the identified transmembrane cargo proteins, 15 were predicted to show plasma membrane localizations, 4 were predicted to show Golgi localizations, and 4 were predicted to show endosome or lysosome localizations (Fig. 3*F* and Dataset S2, Sheet 3).

### ATRAP Is an AP-4 Cargo.

**Fig. 4.**
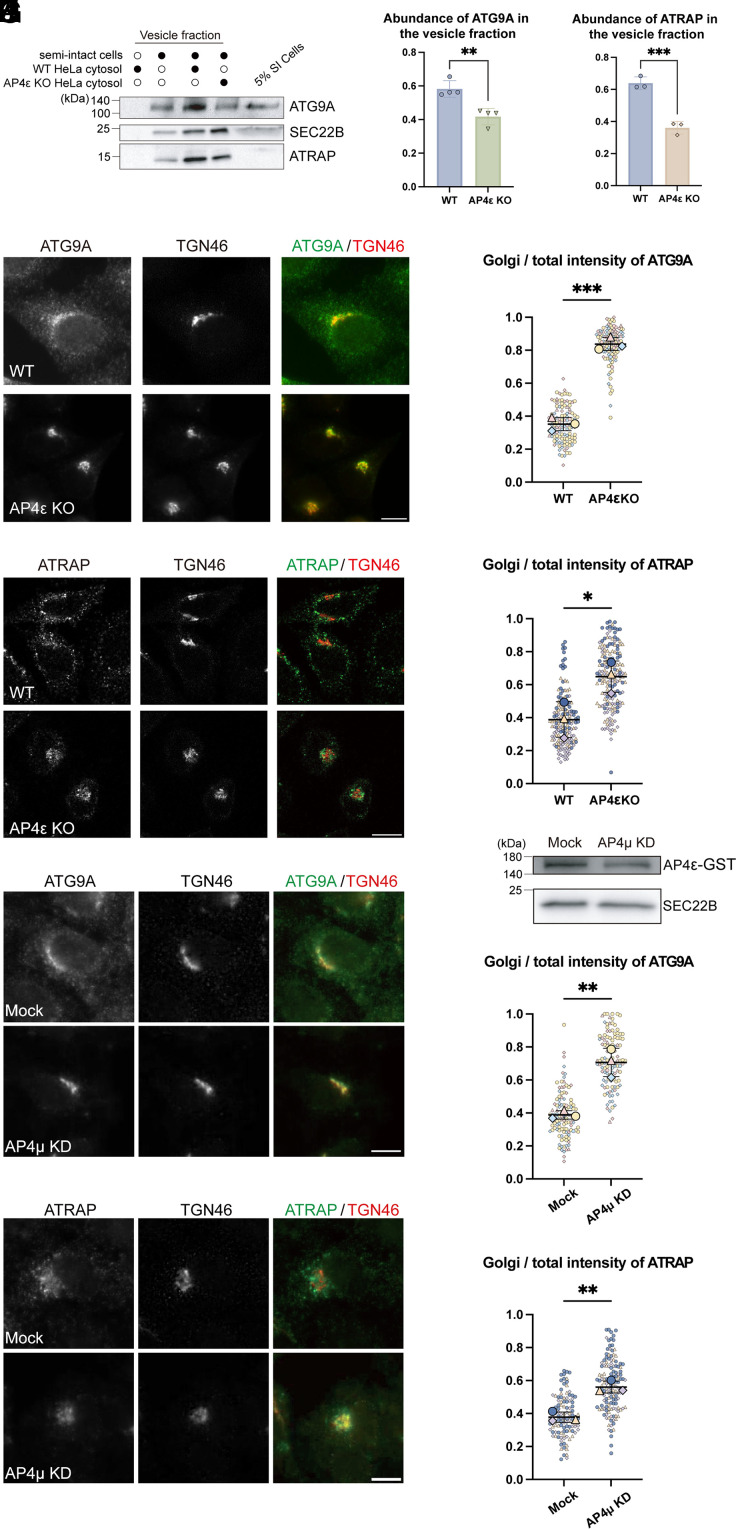
AP-4 mediates the packaging of ATRAP into TGN-derived vesicles. (*A*–*C*) The vesicle formation assay was performed. The vesicle fraction was then analyzed by immunoblot (*A*). Abundances of ATG9A and ATRAP normalized to the abundance of SEC22B were quantified (*B* and *C*, mean ± SD, n ≥ 3). The sum of the value in both experimental groups was normalized to 1 in each replicated experiment. (*D*–*G*) The localizations of ATG9A (*D*) or ATRAP (*F*) in WT or AP4ε KO HeLa at the steady state were analyzed. The ratio of above-threshold fluorescence intensity of ATG9A or ATRAP in the Golgi region to that in the entire cell was quantified and presented using SuperPlot (*E* and *G*). Large symbols represent the mean, and small symbols represent individual cells from each experiment (30 to 68 cells quantified per group in each replicate). Experiments are color- and shape-coded. Horizontal lines indicate the mean ± SD of the means from three independent experiments. (*H*–*L*) WT HeLa cells were transfected with control siRNA (Mock) or siRNA against AP4μ (AP4μ KD). On Day 3 after knockdown, cells were lysed for immunoblot analysis (*H*) or fixed for immunofluorescent assays (*I* and *K*). To measure the abundance of AP-4 (*H*), cells were transfected with four subunits of AP-4 including AP4ε-GST following siRNA transfection. The above-threshold fluorescent intensity of ATG9A or ATRAP at the TGN area over that in the total cell area was quantified (*J* and *L*), with 30 to 69 cells quantified per group in each replicate. (Scale bar, 10 μm.) Statistical significance was calculated using unpaired Student’s *t* test; ***P < 0.001; **P < 0.01; *P < 0.05. SI cells, semi-intact cells.

Among the identified AP-4 cargo clients, we focused on the type-1 angiotensin II receptor-associated protein (ATRAP/AGTRAP), which is predicted to localize at the Golgi and plasma membrane. ATRAP levels showed a fold change >1.3 between WT and AP4ε KO HeLa group but <1 between WT and AP1γ1 KO group (Fig. 3*G* and Dataset S2, Sheet 2), suggesting that its TGN export depends on AP-4 but not AP-1. A similar trend was observed for two known AP-4 clients, ATG9A and DAGLB (Fig. 3*G*). To further explore whether AP-4 mediates the TGN export of ATRAP, we conducted vesicle formation assays and analyzed the vesicle fractions via immunoblotting. The results were consistent with the proteomic data, showing a marked reduction in the abundances of ATRAP and ATG9A in the vesicle fractions when the vesicle formation assay was performed using cytosol from AP4ε KO cells compared to WT cytosol (Fig. 4 *A*–*C*). Consistent with a previous report (11), KO of AP4ε in HeLa cells led to a reduction in cytoplasmic ATG9A and accumulation at the TGN (Fig. 4*D*). Quantification analysis indicates that the ratio of above-threshold fluorescent intensity of ATG9A in the Golgi area over that in the total cell area was significantly higher in AP4ε KO cells than in WT cells (Fig. 4*E*). ATRAP appeared in the juxtanuclear area and as punctate structures at the cell periphery in WT HeLa cells (Fig. 4*F*). By contrast, ATRAP predominantly accumulated in the juxtanuclear Golgi region in AP4ε KO cells (Fig. 4 *F*–*G*). We then performed knockdown analysis and found that knockdown of AP4μ (Fig. 4*H*) also caused a significantly enhanced accumulations of ATG9A (Fig. 4 *I* and *J*) and ATRAP (Fig. 4 *K* and *L*) at the Golgi area. These findings collectively suggest that AP-4 plays a critical role in facilitating the efficient export of ATRAP from the TGN.

### AP-4 Interacts With the Tyrosine Motif of ATRAP, and This Interaction Is Important for TGN Export of ATRAP.

**Fig. 5.**
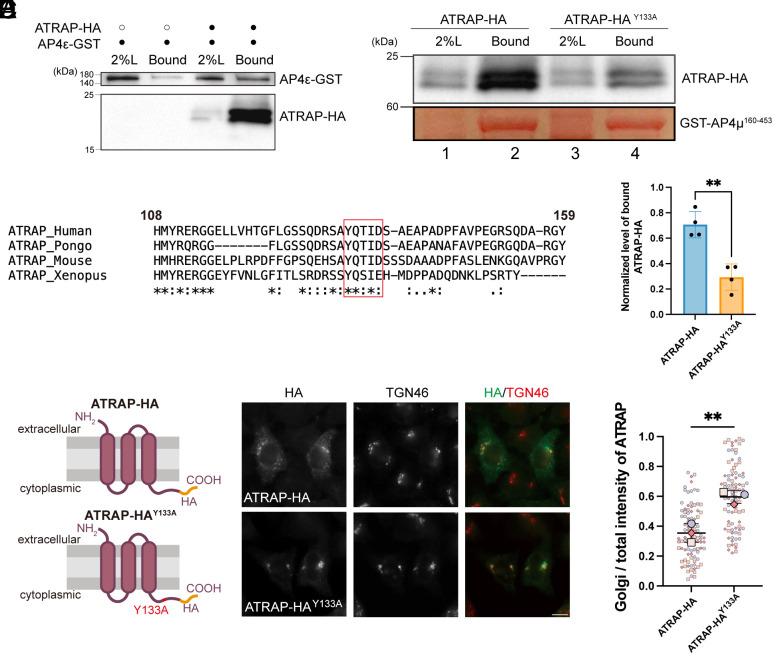
The tyrosine motif of ATRAP is important for its TGN export. (*A*) Co-IP was performed using lysates from HEK293T cells expressing the indicated constructs. The bound proteins were analyzed by immunoblot. (*B*) Sequence alignment of ATRAP across species demonstrating a conserved YXXΦ [E/D] motif (red) in its cytosolic tail (aa 108 to 159). (*C* and *D*) Purified GST-AP4μ^160–453^ was incubated with lysates from HEK293T cells transfected with ATRAP-HA and ATRAP-HA^Y133A^. After incubation, the bound proteins were analyzed by immunoblot (*C*), and the abundance of bound proteins normalized to the 2% loading was quantified (*D*, n = 4, mean ± SD). The sum of the value in both experimental groups was normalized to 1 in each replicated experiment. (*E* and *F*) WT HeLa cells were transfected with plasmids encoding ATRAP-HA or ATRAP-HA^Y133A^. On day 1 after transfection, the localizations of the indicated proteins were analyzed by immunofluorescence (*E*). The above-threshold fluorescent intensity of ATRAP-HA or ATRAP-HA^Y133A^ at the Golgi area over that in the total cell area was quantified (*F*), with 28 to 33 cells quantified per group in each replicate. (Scale bar, 10 μm.) Statistical significance was calculated using an unpaired *t* test; **P < 0.01.

Coimmunoprecipitation experiments demonstrated that AP4ε-GST binds to ATRAP-HA (Fig. 5*A*), suggesting that ATRAP interacts with AP-4. Notably, AP-4 has been previously shown to recognize the YXXΦ[E/D] motif in the cytosolic tails of specific proteins, such as ATG9A (11), and ATRAP contains a similar and conserved tyrosine motif (Fig. 5*B*). To investigate the importance of this motif, we employed GST pull-down assays, which showed that the purified GST-tagged C-terminal fragment of AP4μ (AP4μ^160–453^) binds to ATRAP-HA (Fig. 5*C*). This interaction was significantly reduced when using a mutant form of ATRAP-HA, where the critical tyrosine was replaced with alanine (ATRAP^Y133A^-HA) (Fig. 5*C*, compare lanes 2 and 4, and quantification in Fig. 5*D*). Further immunofluorescence analysis revealed that while the wild-type ATRAP-HA partially localized to the TGN and appeared in punctate structures around the cell periphery, the ATRAP^Y133A^-HA mutant predominantly accumulated at the TGN, when expressed at low levels (Fig. 5*E*). Quantitative analysis showed that the ratio of above-threshold fluorescent intensity of ATRAP in the Golgi region relative to the total cell area was significantly higher for the tyrosine mutant compared to the wild-type protein (Fig. 5*F*). These results highlight the essential role of the tyrosine motif in facilitating the interaction between AP-4 and ATRAP, which is crucial for the efficient export of ATRAP from the TGN.

### Identification of Cytosolic Proteins Mediating the Trafficking of AP-4 Cargos.

The AP-4-mediated TGN export is believed to operate independently of clathrin (19), pointing toward the involvement of unidentified accessory factors that assist AP-4 in vesicle formation at the TGN. Analysis of proteins enriched in the WT group compared to the AP4ε KO group revealed several cytosolic proteins, predicted by Uniprot to localize at the Golgi or endosomes. These cytosolic factors potentially serve as AP-4 accessory factors (Dataset S2, Sheet 4). When the vesicle formation assay was performed using AP4ε KO cytosol, the accessory factors are proposed to be failed to be recruited to vesicle membranes, resulting in reduced abundance. We focused our following analysis on two of the hits: PRRC1 and WD repeat-containing protein 44 (WDR44) (Fig. 3*E*).

**Fig. 6.**
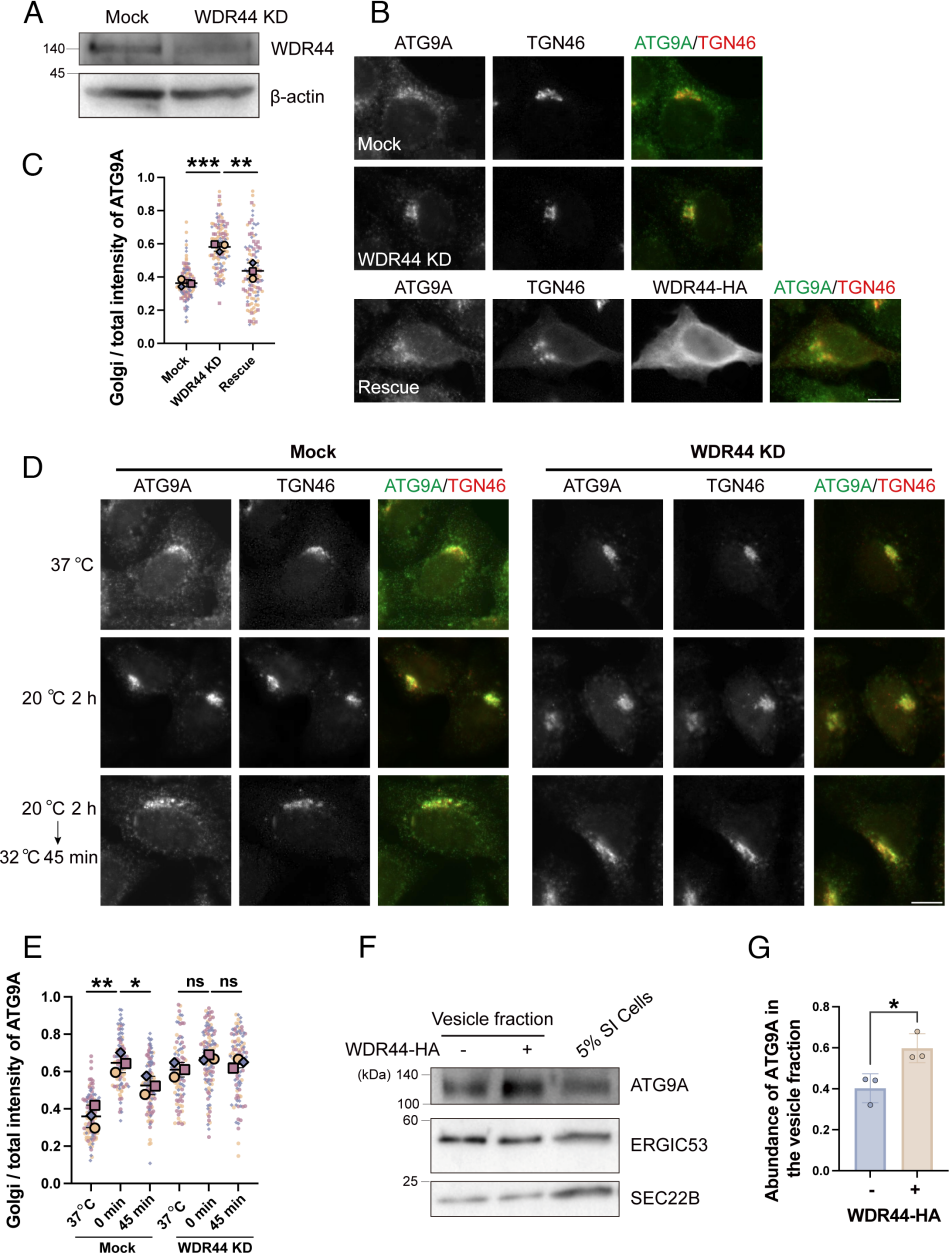
WDR44 mediates TGN export of AP-4 cargo ATG9A. (*A*–*C*) WT HeLa cells were transfected with control siRNA (Mock) or siRNA against WDR44 (WDR44 KD). At 48 h after transfection, cells were lysed for immunoblot analysis (*A*) or fixed and stained with the indicated antibodies (*B*). Golgi/total above-threshold fluorescent intensity of ATG9A was quantified (*C*), with 30 to 51 cells quantified per group in each replicate. (*D* and *E*) Mock or WDR44 KD HeLa cells were incubated at 20 °C for 2 h, followed by temperature shifting to 32 °C for 0 or 45 min and the localizations of the indicated proteins were analyzed (*D*). Golgi/total above-threshold fluorescent intensity of ATG9A was quantified (*E*), with 30 to 46 cells quantified per group in each replicate. (*F* and *G*) The vesicle formation assay was performed using cytosol prepared from WT HeLa cells or WT HeLa cells overexpressing WDR44-HA. The vesicle fractions were analyzed by immunoblot (*F*). The abundances of ATG9A in the vesicle fractions were quantified (*G*, n = 3, mean ± SD). The sum of the value in both experimental groups was normalized to 1 in each replicated experiment. (Scale bar, 10 μm.) Statistical significance was determined using an unpaired *t* test; ***P < 0.001; **P < 0.01; *P < 0.05; ns, no significant. SI cells, semi-intact cells.

WDR44 is a WD repeat-containing protein. WD repeat-containing proteins are involved in various cellular processes, including the assembly of protein complexes (20). To explore the role of WDR44 in TGN sorting, we performed siRNA knockdown in WT HeLa cells (Fig. 6*A*). WDR44 knockdown led to increased ATG9A accumulation at the Golgi compared to control siRNA (Fig. 6*B*). Quantification showed a significant rise in the ratio of above-threshold fluorescent intensity of ATG9A in the Golgi area over that in the total cell area in WDR44 knockdown cells (Fig. 6*C*). We then generated an HA-tagged, siRNA-resistant WDR44 construct (WDR44-HA), which localized to the cytoplasm, consistent with prior findings in HeLa cells (21). Notably, expressing this construct rescued the ATG9A trafficking defect (Fig. 6 *B* and *C*).

We also conducted a temperature shift assay to evaluate the role of WDR44 in TGN export. In cells transfected with control siRNA, the Golgi-localized ATG9A was significantly increased after 2 h at 20 °C and was partially released upon shifting to 32 °C (Fig. 6 *D* and *E*). Notably, while WDR44 knockdown increased Golgi-localized ATG9A under normal conditions (Fig. 6 *B* and *C*), the 20 °C block failed to cause additional accumulation in these cells (Fig. 6 *D* and *E*). Furthermore, the accumulated ATG9A in WDR44 knockdown cells showed impaired release upon temperature shift to 32 °C (Fig. 6 *D* and *E*). Additionally, in vesicle formation assays, the presence of WDR44-HA in the cytosol led to a significantly increased abundances of ATG9A in vesicle fractions, whereas the abundances of ERGIC53 and SEC22B remained unchanged (Fig. 6 *F* and *G*). Collectively, these observations suggest that WDR44 plays a crucial role in TGN export of ATG9A.

**Fig. 7.**
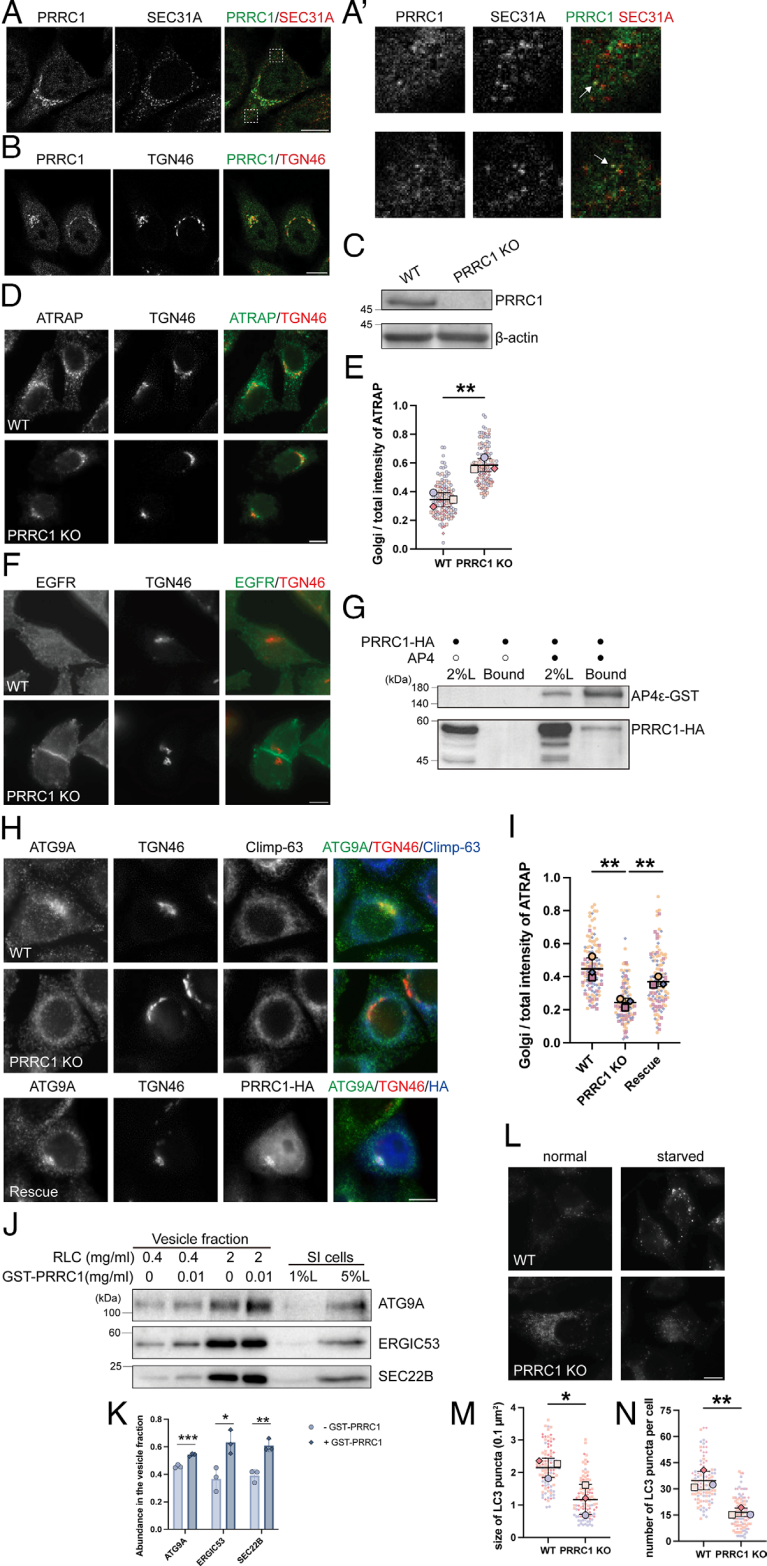
PRRC1 is important for the trafficking of AP-4 cargo clients, ATG9A and ATRAP. (*A* and *B*) The localizations of the indicated proteins were analyzed by immunofluorescence. The magnified views of the indicated areas in panel *A* were shown in panel *A*′. Arrows highlight representative puncta where PRRC1 and SEC31A signals overlap. (*C*) The cell lysates of WT or PRRC1 KO HeLa cells were analyzed by immunoblot. (*D*–*F*) The localizations of ATRAP (*D*) and EGFR (*F*) in WT or PRRC1 KO HeLa were analyzed at the steady state. The Golgi/total above-threshold fluorescent intensity of ATRAP was quantified (*E*), with 30 to 51 cells quantified per group in each replicate. (*G*) Co-IP was performed using lysates from HEK293T cells expressing the indicated constructs. The bound proteins were analyzed by immunoblot. (*H* and *I*) The localizations of ATG9A in WT, PRRC1 KO, or PRRC1 KO cells overexpressing PRRC1-HA were analyzed (*H*). Golgi/total above-threshold fluorescent intensity of ATG9A was quantified (*I*), with 30 to 56 cells quantified per group in each replicate. (*J* and *K*) The vesicle formation assay was performed using the indicated reagents. The vesicle fractions were analyzed by immunoblot (*J*). Abundance of indicated proteins in the vesicle fraction under 0.4 mg/ml RLC was quantified (*K*, n = 3, mean ± SD). For each replicated experiment, the sum of the abundance of each cargo protein was normalized to 1. (*L*–*N*) WT or PRRC1 KO HeLa cells were incubated in EBSS for 0 min or 60 min. After incubation, the localizations of LC3 were analyzed (*L*). The average size (*M*) and number (*N*) of LC3 in starved cells were quantified using ImageJ with the Analyze Particles function, with 30 to 38 cells quantified per group in each replicate. (Scale bar, 10 μm.) Statistical significance was determined using an unpaired *t* test; ***P < 0.001; **P < 0.01; *P < 0.05. RLC, rat liver cytosol; SI cells, semi-intact cells.

PRRC1, a proline-rich and coiled-coil-containing protein, is shown to interact with the inner COPII coat, regulating the membrane association of COPII (17). We found that endogenous PRRC1 exhibited punctate localization at the cell periphery, where it colocalized with a subpopulation of the punctate structures of COPII marker SEC31A (Fig. 7 *A* and *A*′), as well as in the juxtanuclear Golgi region (Fig. 7*B*). To test whether PRRC1 regulates AP-4-mediated TGN export, we generated a PRRC1 KO HeLa cell line (Fig. 7*C*) and assessed the localization of two AP-4 cargos, ATG9A and ATRAP, in both WT and PRRC1 KO HeLa cells. In PRRC1 KO cells, ATRAP was predominantly localized at the TGN without the typical peripheral punctate structures observed in WT cells, indicating a disruption in its TGN export (Fig. 7*D*). Quantification analysis indicates that the ratio of above-threshold fluorescent intensity of ATRAP in the Golgi area over that in the total cell area was significantly increased in PRRC1 KO cells than in WT cells (Fig. 7*E*). By contrast, the localization of EGFR, an AP-1 cargo (22), remained unaffected at the cell surface in both WT and PRRC1 KO cells (Fig. 7*F*), suggesting that the influence of PRRC1 on TGN export is cargo-specific. Furthermore, coimmunoprecipitation experiments suggest AP-4 interacts with PRRC1 (Fig. 7*G*).

Intriguingly, under the same experimental conditions, ATG9A displayed a distinct localization pattern. In WT HeLa cells, ATG9A exhibited juxtanuclear structures and cytoplasmic puncta (Fig. 7*H*). However, in PRRC1 KO HeLa cells, ATG9A was mainly localized to the ER, as indicated by its colocalization with the ER marker Climp-63 (Fig. 7*H*). Quantification analysis indicates that the ratio of above-threshold fluorescent intensity of ATG9A in the Golgi area over that in the total cell area was significantly decreased in PRRC1 KO cells than in WT cells (Fig. 7*I*). This defect was rescued by overexpressing PRRC1-HA (Fig. 7 *H* and *I*). These analyses indicate that PRRC1 plays an important role in the ER export of ATG9A, acting upstream of its export from the TGN. Additionally, purified PRRC1 significantly enhanced the efficiency of packaging of ATG9A into vesicles (Fig. 7 *J* and *K*). The efficiency of packaging of COPII cargo, ERGIC53 and SEC22B, was also enhanced by purified PRRC1 when the assay was performed using a lower concentration of rat liver cytosol (Fig. 7 *J* and *K*).

Given the involvement of ATG9A in autophagy, we assessed the localization of LC3, a marker for autophagosomes, in both WT and PRRC1 KO cells under normal and starvation-induced conditions. While punctate structures of LC3 were formed in starved WT cells, indicating the activation of autophagy (Fig. 7*L*), LC3 appeared more dispersed in PRRC1 KO cells under normal conditions and failed to form the characteristic puncta observed in WT cells during starvation (Fig. 7 *L*–*N*). These findings underscore the importance of PRRC1 in the trafficking of AP-4 cargos, specifically ATG9A and ATRAP. Moreover, they highlight PRRC1’s multifunctional role in regulating different cargo proteins, affecting not only Golgi-to-cell surface transport but also autophagy.

## Discussion

APs play critical roles in mediating protein sorting at the TGN. In this study, we employed a vesicle formation assay using cytosol prepared from WT cells or from cells lacking AP-1 or AP-4 subunits. This approach enabled us to identify potential cargo clients of AP-1 and AP-4. Biochemical validation revealed CAB45 as an AP-1 cargo. CAB45 primarily localizes to the TGN lumen, where it binds a subset of soluble cargo molecules in a Ca^2+^-dependent manner and facilitates their sorting out of the TGN (1). However, as a soluble protein, the mechanism by which CAB45 is incorporated into vesicles at the TGN remains unclear. Our in vitro vesicle formation assays, combined with genetic perturbations in intact cells, reveal that AP-1 is essential for CAB45 export from the TGN. These findings suggest that AP-1 acts as the cytosolic factor responsible for directing CAB45 packaging into transport carriers. We propose that an unidentified transmembrane receptor may link CAB45 to the AP-1 machinery, enabling its selective enrichment in vesicles. Based on our findings, CAB45 client cargoes should also require AP-1 for export from the TGN. However, our mass spectrometry analysis did not detect established CAB45 cargoes such as LysC and COMP, likely due to their low abundance in the vesicle fraction or their loss during trypsinization.

Additionally, we identified ATRAP as an AP-4 cargo, with its interaction dependent on a tyrosine-based sorting motif in its cytosolic domain. ATRAP was initially characterized as an angiotensin II type 1 receptor (AT1R)-binding protein that promotes AT1R internalization and suppresses AT1R-mediated cell proliferation in vascular smooth muscle cells (23, 24). Beyond its role in AT1R signaling, ATRAP interacts with transferrin receptor 1 (TfR1) to regulate its internalization, thereby influencing iron metabolism and oxidative stress responses (25). Notably, ATRAP deficiency exacerbates kidney fibrosis in aged mice, independent of angiotensin signaling (26), highlighting its broader implications in cellular homeostasis and disease. Our results demonstrate that ATRAP packaging into TGN-derived vesicles is AP-4-dependent, implicating its potential role in AP-4 deficiency syndrome.

In addition to identifying cargo proteins, our approach also uncovered accessory factors involved in protein sorting at the TGN. Traditional detergent-based binding assays often fail to capture protein interactions dependent on lipid bilayers. In contrast, our vesicle formation assay preserves the lipid bilayer environment of the ER and Golgi, enabling the identification of membrane-associated binding partners. Through this approach, we identified WDR44 and PRRC1 as cytosolic factors involved in AP-4-mediated cargo sorting at the TGN.

WDR44, a downstream effector of Rab11, binds GTP-bound Rab11 and participates in Rab11-dependent vesicle recycling (27). Rab11 and its effectors, including WDR44, play roles in ciliogenesis (28). WDR44 forms tubules in a Rab11-, Rab8-, and Rab10-dependent manner and is involved in the export of proteins sensitive to ER stress (29). WDR44 also interacts with the COPII outer coat protein Sec13, implicating its role in vesicle trafficking (21). Our findings suggest that WDR44 promotes the TGN export of ATG9A, highlighting its functional diversity within TGN-derived vesicles. PRRC1, another cytosolic protein identified in our study, showed a dual localization at the ER exit sites and the Golgi (Fig. 7 *A* and *B*). We previously showed that PRRC1 regulates COPII membrane associations and ER-to-Golgi trafficking (17). Here, we demonstrate that PRRC1 interacts with AP-4 and differentially regulates the trafficking of AP-4 clients ATG9A and ATRAP, suggesting its complex roles in vesicle trafficking. These findings underscore the utility of the vesicle formation assay combined with proteomic analysis in identifying accessory proteins that regulate vesicle trafficking.

WDR44 contains WD40 repeats, which form a β-propeller structure essential for protein–protein interactions. This structural feature is critical for the assembly of coat proteins in COPII and clathrin-coated vesicles (20), suggesting that WDR44 may play a role in AP-4 coat assembly. PRRC1, on the other hand, contains proline-rich domains, which are known to facilitate protein–protein interactions, provide structural flexibility, and recruit components of the vesicle formation machinery. While PRRC1’s role in AP-4 coat assembly remains unclear, its proline-rich domains suggest potential involvement in regulating vesicle trafficking. Further studies are needed to elucidate the precise mechanisms by which WDR44 and PRRC1 contribute to AP-4-mediated vesicle formation.

## Materials and Methods

### Constructs, Reagents, Cell Culture, Transfection, and Immunofluorescent Assays.

Cell lines, plasmids, siRNAs, antibodies, cell culture, immunofluorescence, and transfection were described in the *SI Appendix*.

### Protein Purification, Immunoprecipitation, and GST Pull Down.

Purification of GST-tagged protein was performed as described (30). Immunopreicipitation and GST pulldown experiment was performed as described previously (31, 32).

### Cytosol Preparation and In Vitro Vesicle Formation Assay.

Mammalian cell cytosol preparation was described in the *SI Appendix*. In vitro vesicle formation assay was performed as described previously (17, 33, 34).

### Sample Preparation for Label-Free Quantitative Mass Spectrometry (MS) Analysis and MS Data Analysis.

These procedures were described in the *SI Appendix*.

### Quantification and Statistical Analyses.

These procedures were described in the *SI Appendix*.

## Supplementary Material

Appendix 01 (PDF)

Dataset S01 (XLSX)

Dataset S02 (XLSX)

## Data Availability

All study data are included in the article and/or supporting information.
