# Supplementary material for: Uncovering cargo clients and accessory factors of AP-1 and AP-4 through vesicle proteomics
Source: Proc Natl Acad Sci U S A. 2025 Oct 1;122(40):e2508961122. doi: 10.1073/pnas.2508961122 (PMC12519218; doi:10.1073/pnas.2508961122)
Supplement: Supplementary file 1 — Appendix 01 (PDF) [file pnas.2508961122.sapp.pdf]

Supplemental Information (SI) Appendix

Uncovering Cargo Clients and Accessory Factors of AP-1 and AP-4 through Vesicle Proteomics

Ziqing Peng[1], Jingran Fan[1], Yang Liu[1], Qinyu Jia[2,3], Junkun An[1], Jianying Wang[2,3], Yan Huang[1], Zhong-Ping Yao[2,3,*], Yusong Guo[1,4,*]

1. Division of Life Science and State Key Laboratory of Molecular Neuroscience, The Hong Kong University of Science and Technology, Hong Kong
2. State Key Laboratory of Chemical Biology and Drug Discovery, Research Institute for Future Food, Research Centre for Chinese Medicine Innovation, and Department of Applied Biology and Chemical Technology, The Hong Kong Polytechnic University, Hung Hom, Kowloon, Hong Kong Special Administrative Region, China
3. State Key Laboratory of Chinese Medicine and Molecular Pharmacology (Incubation), and Shenzhen Key Laboratory of Food Biological Safety Control, Hong Kong Polytechnic University Shenzhen Research Institute, Shenzhen 518057, China
4. Thrust of Bioscience and Biomedical Engineering, Hong Kong University of Science and Technology, Guangzhou 511453, China

*, Corresponding author: guoyusong@ust.hk; zhongping.yao@polyu.edu.hk

Includes:

SI Materials and Methods

SI Materials and Methods

Cell culture

HEK293T and HeLa cell lines were kindly provided by the University of California-Berkeley Cell Culture Facility. AP1γ1 KO HeLa and AP4ε KO HeLa cell lines were generously provided by Dr. Juan S. Bonifacino's lab (NIH). For generating CRISPR-Cas9–mediated PRRC1 KO HeLa cells, guide RNAs targeting exon 2 of the PRRC1 gene (5'-TCCACCAAATCCTGCAGGGC-3') were designed using the CRISPR design tool (http://crispor.tefor.net/) and subsequently cloned into AAV9-U6-DsRed plasmids. Multiple single–cell-derived KO clones were obtained through fluorescence-activated cell sorting and screened for gene disruption by Western blotting. Clonal cell lines were validated by Sanger sequencing of the genomic PCR at the target site. All these cell lines were cultured in Dulbecco's Modified Eagle's Medium (DMEM) (Gibco™) with 10% Fetal Bovine Serum (FBS) (Gibco™) and 1% penicillin/streptomycin (Invitrogen). For experiments requiring temperature shifts, cells were incubated in Optimized Minimum Essential Medium (Opti-MEM) (Gibco™) containing 10% FBS at 20°C for 2 hours to block cargo proteins at the TGN. The temperature was then raised to 32°C to release the cargo for subsequent immunofluorescent assays.

Plasmids, siRNAs and antibodies

The CAB45-HA, ATRAP-HA, WDR44-HA and PRRC1-HA were ordered from BGI (Beijing, China). The construct encoding ATRAP-HA^{Y133A} was generated by the QuickChange site-directed mutagenesis procedure. The plasmids encoding AP4μ, AP4σ, AP4ε-GST and 2-Strep-1-Flag-AP4β-Maltose binding protein were kindly provided by Dr. Juan S. Bonifacino's lab (NIH) (1).

The plasmids encoding GST-AP4μ$^{160-453}$, GST-PRRC1 and AP1γ1-FLAG were generated by standard molecular cloning procedures. The plasmid encoding HA-Vangl2 was described previously (2). The siRNAs used in this study were purchased from Ribo-bio (Guangzhou, China). The target sequence of the siRNA against AP1G1 is TAGCACAGGTTGCCACTAA. The target sequence of the siRNA against AP4M1 is GGACGAATTTGAGTCTCAT. The target sequence of the siRNA against WDR44 is GAACCTGGATACTGGAGAA.

Polyclonal rabbit antibodies against SEC23A, SEC22B and ERGIC53 for immunoblot analyses were gifts from Dr. Randy Schekman's lab (UC Berkeley). The commercial antibodies were: Rabbit anti-HA (Cell signaling technology, number 3724S); Mouse anti-HA (Biolegend, number 901501); Rabbit anti-CAB45 (Invitrogen, number PA5-120363); Rabbit anti-VTI1B (Proteintech, number 14495-1-AP); Sheep anti-TGN46 (AbD Serotec, number AHP500G); Rabbit anti-ATG9A (Cell signaling technology, number 13509); Rabbit anti-ATRAP (Invitrogen, number PA5-60617); Rabbit anti-PRRC1 (Bethyl, number A305-783); Rabbit anti-PRRC1 (Boster, number A15083-1); Goat anti-GST (GE Healthcare, number 27-4577-01); Mouse anti-β actin (Proteintech, number 60008-1-Ig); Mouse anti-AP1γ1 (Sigma,

number A4200); Rabbit anti-WDR44 (NOVUS, NBP2-56672); Mouse anti-EGFR (Santa Cruz, number 365829); Mouse anti-SEC31A (BD, number 612350), Mouse anti-Climp63 (Enzo, number ENZ-ABS669); Rabbit anti-SEC31A (Bethyl, number A302-336A).

Cytosol preparation and in vitro vesicle formation assay

To prepare mammalian cell cytosol, WT HeLa, AP1γ1KO HeLa, and AP4ε KO HeLa cells were centrifuged at 300 g for 3 minutes. After centrifugation, the cell pellets were suspended in ice-cold buffer E (50 mM Hepes, 250 mM sorbitol, 70 mM potassium acetate (KOAc), 5 mM EGTA and 0.5 mM magnesium acetate ($Mg(OAc)_2$), pH 7.2) containing 5 mM dithiothreitol (DTT) and 1x protease inhibitor (PI) and homogenized using a needle. The resulting homogenate was centrifuged twice at 16,900 g for 10 minutes to eliminate cell debris. The supernatant was further centrifuged at 100,000 g in an S55S rotor (Hitachi) for 1 hour, and the concentration of cytosolic protein fractions was determined using Bio-Rad protein assay dye reagent concentrate according to the manufacturer's instructions.

The *in vitro* vesicle formation assay was performed as described previously (3). Cells were collected and permeabilized in ice-cold KOAc buffer (110 mM KOAc, 2.5 mM $Mg(OAc)_2$, 20 mM Hepes, pH 7.2) containing 40 μg/ml digitonin for 5 minutes on ice. The permeabilized cells were then collected by centrifugation at 300 g for 3 minutes at 4°C, washed, and resuspended in KOAc buffer. The cells were incubated at 32°C with 2 mg/ml cytosol, 200 μM GTP (Wako), and ATP regeneration system (ATPrS, containing 40 mM creatine phosphate (Roche), 0.2 mg/ml creatine phosphokinase (Roche), and 1 mM ATP (Sigma)) for 60 minutes. After incubation, the reaction mixture was centrifuged at 16,900 g for 20 minutes at 4°C to remove ER and Golgi membranes, nuclei, and other cell debris. The supernatant containing the released vesicles was resuspended in 35% Opti-Prep™ Density Gradient Medium (Sigma-Aldrich) and overlaid with 30% Opti-Prep and KOAc buffer on the top. The Opti-Prep gradients were centrifuged at 55,000 rpm in an S55S rotor at 4°C for 1.5 hours. Following centrifugation, the top 200 μl of the gradient was collected, mixed with 300 μl KOAc buffer, and centrifuged at 55,000 rpm in an S120-AT3 rotor at 4°C for 30 minutes. The resulting vesicle pellets were resuspended and analyzed using Western blot and mass spectrometry.

Transfection and immunofluorescent assays

The DNA constructs and siRNAs were transfected into cells by lipofectamine 2000 (Invitrogen) for WT or KO HeLa cell lines or by PEI for HEK293T, as described in the manual provided by Invitrogen. Each siRNA was used at a final working concentration of 40 nM.

For immunofluorescence experiments, cells were seeded onto 13 mm coverslips. Cells were fixed by incubation in 4% paraformaldehyde (PFA) (Sigma-Aldrich) in phosphate-buffered saline (PBS) (137 mM NaCl, 2.8 mM KCl, 10 mM Na_2HPO_4, 1.8 mM KH_2PO_4, pH 7.4) at room temperature for 15 minutes. After fixation, cells were permeabilized and blocked using blocking buffer (2.5% FBS, 0.1% Triton X-

100, and 200 mM glycine in PBS) for 30 minutes at room temperature. The cells were then incubated with primary antibodies in blocking buffer at room temperature for 30 minutes, washed three times with PBS, and incubated with specified secondary antibodies in blocking buffer for 30 minutes at room temperature. Secondary antibodies were Alexa Fluor®488 or 568 or 647-coupled donkey antibodies targeting rabbit, mouse, or sheep IgG (Invitrogen). After washing the cells three times with PBS, they were mounted onto slides using a homemade MOWIOL mounting medium, which is a solution of Mowiol containing 2.5% 1,4-diazobicyclo-[2.2.2]-octane (DABCO, Sigma, D2522) (preparation as described here: https://www.nichd.nih.gov/about/org/dir/other-facilities/cores/microscopyandimaging/support/MOWIOL). For LC3 staining, the cells were permeabilized with 50 μg/ml digitonin and stained as described previously (4). Images were acquired using a Nikon Fluorescence Microscope with filter sets (FITC, Alexa 568, and Cy5) or a Leica SP8 Confocal Microscope equipped with 488 nm and 552 nm lasers. Detailed specifications for these instruments can be found at https://www.biocrf.ust.hk/microscopy. The confocal images were captured using the line sequential scanning acquisition mode. Images were analyzed using Fiji software.

Quantification and statistical analysis

For Western blot quantification, the sum of the intensity of the two experimental groups (e.g., the WT group and the AP1γ1 KO group in Figure 2B) in each replicate experiment was normalized to 1. This approach places all biological replicates on a common scale while preserving the relative statistical distribution across independent experiments.

To quantify the percentage of cells exhibiting punctate CAB45-HA localization, cells from all experimental groups were analyzed under identical exposure conditions. A fixed threshold was uniformly applied to assess CAB45-HA intracellular localization in each cell. Cells displaying above-threshold puncta outside the Golgi region were classified as exhibiting punctate CAB45-HA localization. Quantification of the number and size of LC3 puncta using ImageJ with the Analyze Particles function. A fixed threshold was used in each of the independent replicate.

For Golgi/total fluorescence intensity measurements, single optical sections were analyzed using ImageJ. Individual cells were manually selected with the free-hand tool, and the Golgi area was identified based on the TGN46 channel. The "Moments" auto threshold method was applied to effectively capture the TGN46 signal. Above-threshold fluorescence areas were then selected and saved. The same "Moments" auto threshold method was applied to the cargo channels (ATG9A or ATRAP) to measure the total above-threshold fluorescence intensity, quantifying both Golgi-localized and whole-cell cargo protein signals with a fixed threshold per cell. This auto threshold method was applied to minimize bias from visual judgment. The "Moments" method available in ImageJ effectively captures true signals while filtering out background noise, as confirmed by comparisons with the original grayscale images.

Results were presented using Superplots (5) in Figures 4E, 4G, 4J, 4L, 5F, 6C, 6E, 7E, 7I, 7M, 7N. Briefly, big symbols represent the mean, and small symbols represent individual cells from each experiment. Experiments are color- and shape-coded. Horizontal lines indicate the mean ± SD of the means from three independent experiments. All statistical significance in our work were calculated using an unpaired Student's *t* test based on at least three biological repeats.

Mass spectrometry analysis

Samples for mass spectrometry analysis were prepared as previously described (3). Briefly, the vesicle pellets were resuspended in 0.1% RapiGest in 50 mM triethylammonium bicarbonate. Urea was added to a final concentration of 4 M. The samples were then reduced with 10 mM tris (2-carboxyethyl) phosphine at 37°C for 1 hour and alkylated with 20 mM Iodoacetamide at room temperature for 30 minutes in the dark. To digest the proteins into peptides, sequencing grade modified trypsin (Promega, number V511A) was added, and the reaction mixture was incubated at 37°C for 20 hours. The pH of the digested samples was adjusted to between 2.5 and 3 by adding trifluoroacetic acid (TFA) to break down the RapiGest. Degraded RapiGest was removed by brief centrifugation for 10 minutes at 14,000 g at 4°C. The supernatant was dried using a speed vacuum concentrator. Samples were then dissolved with 0.1% TFA, desalted with activated C18 spin column, and dried again.

The dehydrated peptide samples underwent separation via liquid chromatography. A gradient elution process occurred with a 300 nl/min flow rate and a 90-minute acetonitrile gradient, employing an Ultimate 3000 nanoLC system (Thermo Fisher Scientific, San Jose, CA). Subsequently, an Orbitrap Fusion Lumos Tribrid mass spectrometer (Thermo Fisher Scientific, San Jose, CA) in positive ion mode analyzed the samples. The electrospray ionization (ESI) voltage was established at 2300 KV for ion production, and the ion transfer tube temperature was set at 300°C. High-resolution Orbitrap was utilized for both MS and MS/MS scans, with resolution values of 60K and 15K, respectively. A data-dependent acquisition (DDA) mode operated with a 3-second cycle time. The full MS scan spanned from 400 to 1600 m/z, and sequential MS/MS scans commenced at m/z 110. Lastly, the collision energies were configured at 30%.

In each group, three biological replicates were conducted. Protein identification and quantification were carried out using Proteome Discoverer with the following settings: 1) high false discovery rate (FDR<0.01); 2) unique peptides greater than 1; 3) presence in the wild-type (WT) group as either a peak or high; 4) ratio observed in all three biological replicates; 5) assignment of 0 for no abundance; 6) ratio normalized to the median value; 7) the abundance of each identified protein was first normalized to the median protein abundance within its experimental group. Then, for each replicate, the sum of the normalized values across both experimental groups was rescaled to 600. A Student's *t*-test was used to assess the significance of differences between the two experimental groups, based on the normalized protein abundance values from each biological replicate. A 1.3-fold enrichment cutoff was intentionally

chosen based on the behavior of two established AP-4 cargoes (ATG9A and DAGLB), which similarly show fold changes just above this level.

Protein purification, immunoprecipitation and GST pull down

GST-tagged AP4μ$^{160-453}$, GST-tagged PRRC1 and His-tagged SAR1^{H79G} were purified from *E. coli* BL21. The cells expressing the indicated constructs were grown in lysogeny broth (LB) to an OD_{600} of 0.6-0.8 and then induced with 0.5 mM isopropylβ-D-1-thiogalactopyranoside (IPTG) (Sigma-Aldrich) at 16°C overnight (GST-tagged AP4μ$^{160-453}$) or 25°C overnight (GST-tagged PRRC1) or 25°C 4 hours (His-tagged SAR1^{H79G}) to induce protein expression. The cells expressing GST-tagged proteins were lysed on ice for 30 mins with lysis buffer (50 mM Tris, pH 8.0, 5 mM EDTA, 150 mM NaCl, 10% glycerol and 5 mM DTT, 0.5 mg/ml lysozyme, 1x PI). The cells expressing His-tagged SAR1^{H79G} were lysed on ice for 30 mins with 2x PBS containing 10 mM imidazole, 1x PI, 1 mM DTT and 0.5 mg/ml lysozyme. After lysis, 0.5% Triton X-100 was added. The cell lysate was sonicated and then centrifuged at 100,000 g for 10 minutes to remove cell debris. The supernatant was incubated with glutathione agarose beads (Thermo Scientific) or HisPur^{TM} Ni-NTA Resin at 4°C overnight. The beads bearing GST-tagged proteins were washed three times with PDT buffer (PBS containing 1 mM DTT and 0.1% Tween-20) and twice with PD buffer (PBS containing 1mM DTT). The beads bearing GST-PRRC1 were incubated with elution buffer (25 mM glutathione, 50 mM Tris, 250mM KCl, 2 mM DTT, 2x PI, pH 8.0) at 4°C for 4 hours. The beads bearing His-tagged proteins were washed four times with 2x PBS containing 20 mM imidazole, then incubated with elution buffer (250 mM imidazole, 2x PI and 2 mM DTT in KOAc buffer). The eluted proteins were dialyzed against KOAc buffer. The size and concentration of the proteins were verified by SDS-PAGE and Coomassie Blue staining, using different amounts of Bovine serum albumin (BSA) as a standard.

For immunoprecipitation of ATRAP and AP-4, plasmids encoding ATRAP-HA and AP4ε-GST were co-transfected into HEK293T cells. One day after transfection, the cells were harvested and lysed by lysis buffer (KOAc buffer containing 0.5% Triton X-100, 1x PI and 1 mM DTT). The cell lysates were then incubated with beads conjugated with anti-HA antibodies at 4°C for 4 hours. After incubation, the protein-binding beads were washed three times with KOAc buffer containing 0.1% Triton X-100, followed by SDS-PAGE and Western blot analysis. For immunoprecipitation of PRRC1 and AP-4, plasmids encoding PRRC1-HA, AP4μ, AP4σ, AP4ε-GST and Two Strep One Flag-AP4β-Maltose binding protein were co-transfected into HEK293T. One day after transfection, cells were harvested and lysed by lysis buffer (KOAc buffer containing 0.5% Triton X-100, 1x PI and 1mM DTT). Then the cell lysates were incubated with glutathione agarose beads at 4°C for 4 hours. After incubation, the protein-binding beads were washed with KOAc buffer containing 0.1% Triton X-100 for 3 times, followed by SDS-PAGE and Western blot analysis.

For GST pull-down experiment, one day after transfection of plasmids encoding ATRAP-HA or ATRAP-HA^{Y133A}, HEK293T cells were harvested and lysed by lysis buffer (KOAc buffer containing 0.5% Triton X-100, 1x PI and 1mM DTT). Then the cell lysates were incubated with purified GST-AP4μ$^{160-453}$ bound to beads at 4°C for 1.5 hours. Then the beads were washed with KOAc buffer containing 0.1% Triton X-100 for 3 times, followed by Ponceau S (0.5% (w/v) Ponceau S, 1% acetic acid staining) and Western blotting.

SI References

1. R. Mattera, C. D. Williamson, X. Ren, J. S. Bonifacino, The FTS-Hook-FHIP (FHF) complex interacts with AP-4 to mediate perinuclear distribution of AP-4 and its cargo ATG9A. *Mol Biol Cell* **31**, 963-979 (2020).
2. T. Ma *et al.*, A mechanism for differential sorting of the planar cell polarity proteins Frizzled6 and Vangl2 at the trans-Golgi network. *J Biol Chem* **293**, 8410-8427 (2018).
3. Y. Huang *et al.*, An in vitro vesicle formation assay reveals cargo clients and factors that mediate vesicular trafficking. *Proc Natl Acad Sci U S A* **118** (2021).
4. S. Li *et al.*, A new type of ERGIC-ERES membrane contact mediated by TMED9 and SEC12 is required for autophagosome biogenesis. *Cell Res* **32**, 119-138 (2022).
5. S. J. Lord, K. B. Velle, R. D. Mullins, L. K. Fritz-Laylin, SuperPlots: Communicating reproducibility and variability in cell biology. *J Cell Biol* **219** (2020).
